# Admission Cell Free DNA Levels Predict 28-Day Mortality in Patients with Severe Sepsis in Intensive Care

**DOI:** 10.1371/journal.pone.0100514

**Published:** 2014-06-23

**Authors:** Avital Avriel, Maya Paryente Wiessman, Yaniv Almog, Yael Perl, Victor Novack, Ori Galante, Moti Klein, Michael J. Pencina, Amos Douvdevani

**Affiliations:** 1 Pulmonology Institute, Department of Internal Medicine, Soroka University Medical Center and Faculty of Health Sciences, Ben-Gurion University of the Negev, Beer-Sheva, Israel; 2 Clinical Research Center, Soroka University Medical Center and Faculty of Health Sciences, Ben Gurion University of the Negev, Beer-Sheva, Israel; 3 Medical Intensive Care Unit, Soroka University Medical Center and Faculty of Health Sciences, Ben-Gurion University of the Negev, Beer-Sheva, Israel; 4 General Intensive Care Unit, Soroka University Medical Center and Faculty of Health Sciences, Ben-Gurion University of the Negev, Beer-Sheva, Israel; 5 Duke Clinical Research Institute, Durham, North Carolina, United States of America; 6 Clinical Biochemistry and Pharmacology, Soroka University Medical Center and Faculty of Health Sciences, Ben-Gurion University of the Negev, Beer-Sheva, Israel; Institut de Pharmacologie et de Biologie Structurale, France

## Abstract

**Aim:**

The aim of the current study is to assess the mortality prediction accuracy of circulating cell-free DNA (CFD) level at admission measured by a new simplified method.

**Materials and Methods:**

CFD levels were measured by a direct fluorescence assay in severe sepsis patients on intensive care unit (ICU) admission. In-hospital and/or twenty eight day all-cause mortality was the primary outcome.

**Results:**

Out of 108 patients with median APACHE II of 20, 32.4% have died in hospital/or at 28-day. CFD levels were higher in decedents: median 3469.0 vs. 1659 ng/ml, p<0.001. In multivariable model APACHE II score and CFD (quartiles) were significantly associated with the mortality: odds ratio of 1.05, p = 0.049 and 2.57, p<0.001 per quartile respectively. C-statistics for the models was 0.79 for CFD and 0.68 for APACHE II. Integrated discrimination improvement (IDI) analyses showed that CFD and CFD+APACHE II score models had better discriminatory ability than APACHE II score alone.

**Conclusions:**

CFD level assessed by a new, simple fluorometric-assay is an accurate predictor of acute mortality among ICU patients with severe sepsis. Comparison of CFD to APACHE II score and Procalcitonin (PCT), suggests that CFD has the potential to improve clinical decision making.

## Introduction

Decision making process in the setting of the intensive care unit (ICU) is commonly supported by scoring methods such as the Acute Physiology And Chronic Health Evaluation (APACHE) II or the Sequential Organ Failure Assessment (SOFA) score [1], [2]. A novel approach, which incorporates specific blood biomarkers has been put forward to improve the early diagnosis and assessment of patients with sepsis [3]. However, today there is no shelf-ready, accessible serological marker available for the routine clinical practice [4].

Circulating cell-free DNA (CFD), a product of cell necrosis, apoptosis and active secretion, is being investigated as a new reliable marker for assessing prognosis in various pathologies, including cancer, trauma and chronic renal failure (CRF) treated by hemodialysis [5], [6], [7], [8]. Several studies evaluated the prognostic accuracy of CFD for ICU general-patients and septic-patients for death prediction and found good correlation with other outcome predicting scores and markers, such as APACHE, SOFA and CRP level [9], [10], [11], [12], [13], [14]. In a recent publication, Dwivedi et al. found that their CFD assay had a sensitivity of 87.9% and specificity of 93.5% for predicting ICU mortality in patients with severe sepsis- better than multiple organ dysfunction (MOD) and APACHE II scores [14].

Although there is general agreement on the potential value of CFD measurement, to date there is no standard applicable method for routine clinical use. The currently available research methods for CFD measurement require DNA extraction and are labor-intensive. Consequently, CFD measurements are not utilized in the routine patient management. We have developed a novel rapid and direct fluorescent assay for CFD quantification that does not require DNA extraction and PCR amplification, and it has been shown to be inexpensive, accurate and reproducible [15].

Another potentially useful marker for prognostication in patients with severe sepsis is procalcitonin (PCT). PCT was shown to be a relatively reliable method for the outcome prediction in critically ill patients [4], [12], [16].

The aim of the current study is to assess the mortality prediction accuracy of CFD level at admission, measured by a new simplified method, as compared to the conventional clinical score and procalcitonin.

## Methods and Materials

### Population

The study was conducted in Soroka University Medical Center, a tertiary 1000 bed hospital. The research protocol was approved by the “Local Ethics Committee” of the Soroka Medical Center, Beer-Sheva, Israel. Written Informed consent was obtained for patients capable of understanding the study procedures. Institutional review board allowed for an independent physician (not affiliated with the study team) to sign the proxy consent form in a case patient was unconscious (approval number 4636). The study population comprised severe sepsis patients admitted to ICU between March 2009 and 30^th^ of April 2012. We excluded patients less than 18 years of age, pregnant, with CRF, malignancy, and were less than 14 days after surgical procedure (including coronal catheterization) or post trauma.

Enrolled patients were assessed during the first 12 hours of ICU stay. CFD levels were measured within 12 hours from ICU admission. PCT levels were measured upon admission in 70 patients. PCT was determined using a chemiluminescent assay (Liaison Brahms PCT; DiaSorin S.P.A., Saluggia, Italy). We recorded clinical data related to the course of hospitalization, including need for invasive mechanical ventilation, treatment with vasopressors, occurrence of system organ failure and clinical outcomes. Severe sepsis and system organ failure was defined based on the ACCP/SCCM consensus document [17].

### CFD measurements

CFD was detected directly in sera using SYBR Gold Nucleic Acid Gel Stain, (Invitrogen, Paisley, UK) according to the fluorometric method we published [15].

Intra-day coefficient of variation was 16%, 7.9% and 4.8% in the low (383 ng/ml), elevated (1152 ng/ml) and high DNA range (2735 ng/mL), respectively. Day-to-day coefficient of variation was 31%, 6.7% and 8% in the low, elevated and high DNA range, respectively. This method was tested in comparison with the gold standard, QPCR to β-globin and was found to be in good correlation of R^2^ = 0.9987 (p<0.0001) as previously described.[15] The normal cutoff level of 850 ng/ml was established in our previous studies [5], [8], [15], [18].

### Statistical Analysis

In-hospital and twenty eight day all-cause mortality was the study's primary endpoint. Data were summarized using frequency tables, summary statistics, confidence intervals and p-values, as appropriate. The preferred method of analyses for continuous variables was parametric (variables presented as mean ± standard deviation). Not normally distributed variables were presented as median with interquartile range (IQR). Parametric model assumptions were assessed using Normal-plot or Shapiro-Wilks statistic for verification of normality and Levene's test for verification of homogeneity of variances. Non parametric analysis methods were used only if parametric assumptions could not be satisfied (Mann-Whitney test). Categorical variables were tested using Pearson's χ^2^ test for contingency.

Multivariable logistic regression was used to examine the predictive value of the following independent variables on the primary endpoint (death during hospitalization or within 28 day of ICU admission): APACHE II score, quartiles of CFD levels and quartiles of PCT levels. In addition we have assessed c-statistics derived as AUC for ROC curves built on the endpoint probability based on the multivariable logistic regression models inclusive of APACHE-II, CFD, PCT and their combinations. We have used IDI approach to compare the prediction accuracy of the different scores and models [19].

Locally weighted polynomial regression was used to create local regression (LOESS) analysis. Logistic regression models were used to create LOESS analysis for the likelihood of dying as a function of CFD levels at admission adjusted for APACHE-II score.

All statistical tests and/or confidence intervals (CIs) were performed at α = 0.05 (2-sided). P-values are presented after rounding to three decimal places. All statistical analyses were performed using the Statistical Program for Social Sciences (SPSS 18.0 for Windows; SPSS Inc., Chicago, IL, USA).

## Results

### Study Population

One hundred and eight patients were diagnosed with severe sepsis on admission. Out of those, 32.4% (35 patients) have died either during the hospitalization or within 28 days of admission to the ICU.

Table 1 presents clinical and demographic characteristics of the patient population, stratified by 28-day or hospitalization survival status. The majority of the patients were male (71, 65.7%), with mean age being 55.9±17.2 years. Patients, who died, as expected, had higher APACHE II score: median 26 (IQR 15–31) vs. survivors 17 (IQR 11.5–24.5), p = 0.003. Incidence of the different sources of infection was similar between the two groups.

**Table 1:** Patient demographics and clinical data at baseline.

	All Patients with severe sepsis n = 108	Died in hospitalization or within 28 days n = 35	Survivors n = 73	P value
**Age, years** (±SD)	55.88 (±17.2)	60.31 (±15.31)	53.8 (±17.7)	0.063
**Gender** (Male%)	71 (65.7%)	27 (77.1%)	44 (60.3%)	0.084
**APACHE II score** median (IQ range)	20 (12.25–27.75)	26 (15–31)	17 (11.5–24.5)	0.003
**Background Diseases** (%)				
** CIHD**	24 (22.2%)	13 (37.1%)	11 (15.1%)	0.01
** CHF**	8 (7.4%)	7 (20%)	1 (1.4%)	0.001
** CRF**	21 (19.4%)	9 (25.7%)	12 (16.4%)	0.254
** HTN**	47 (43.5%)	14 (40%)	33 (45.2%)	0.61
** DM**	37 (34.3%)	14 (40%)	23 (31.5%)	0.384
** COPD**	7 (6.5%)	5 (14.3%)	2 (2.7%)	0.023
** Creatinine** (±SD, mg/dL)	2.05 (±2.01)	2.2 (±1.75)	1.98 (±2.25)	0.606
** Albumin** (±SD, g/dL)	2.8 (±0.75)	2.34 (±0.57)	2.99 (±0.74)	0.024
** Hemoglobin** (±SD, g/dL)	11.38 (±2.74)	11.48 (±3.14)	11.33 (±2.55)	0.792
** CFD** ng/ml median (IQ range)	1979 (1000.8–3365.5)	3469.0 (1984.0–8833.0)	1659.0 (855.5–2613.5)	<0.001
** PCT** ng/ml median (IQ range)	2.39 (0.39–11.34)	6.49 (0.91–13.87)	1.05 (0.37–7.69)	0.06
** Lactate** mmol/L median (IQ range)	1.4 (0.90–2.35)	2.1 (1.25–4.3)	1.2 (0.90–1.87)	0.007
**Source of sepsis (%)**				0.686
** Lung**	56 (51.9%)	18 (51.4%)	38 (52.1%)	
** UTI**	15 (13.9%)	5 (14.3%)	10 (13.7%)	
** Abdomen**	11 (10.2%)	5 (14.3%)	6 (8.2%)	
** CNS**	6 (5.6%)	0	6 (8.2%)	
** Skin**	4 (3.7%)	2 (5.7%)	2 (2.7%)	
** Blood**	2 (1.9%)	1 (2.9%)	1 (1.4%)	
** Other**	8 (7.4%)	2 (5.7%)	6 (8.2%)	
** unknown**	6 (5.6%)	2 (5.7%)	4 (5.5%)	
**Microorganisms (%)**				0.321
** Gram-negative bacteria**	47 (43.5%)	19 (54.3%)	28 (38.4%)	
** Gram-positive bacteria**	16 (14.8%)	4 (11.4%)	12 (16.4%)	
** Mixed**	10 (9.3%)	4 (11.4%)	6 (8.2%)	
** Unknown**	35 (32.4%)	8 (22.9%)	27 (37%)	

Prevalence of chronic ischemic heart disease (CIHD), congestive heart failure (CHF) and chronic obstructive pulmonary disease (COPD) was higher in patients who did not survive (37.1% vs. 15.1%, 20% vs. 1.4% and 14.3% vs. 2.7% respectively). No significant difference was found in the prevalence of CRF, hypertension (HTN) and diabetes mellitus (DM). Albumin level was significantly lower in patients who did not survive (2.34±0.57 g/dL vs. 2.99±0.74 g/dL, p = 0.024). There was no significant difference in creatinine and hemoglobin levels between patients who survived and patients who did not survive.

### Clinical Course

All non-survivors patients had multi-system organ failure compared with 75.3% of survivors (p = 0.001). Hematologic, liver and CNS failures were significantly more common among patients who did not survive (34.3% vs. 8.2%, p = 0.001, 28.6% vs. 4.1%, p<0.001 and 68.6% vs. 43.8%, p = 0.016 respectively). Respiratory, renal and hemodynamic failure rates were somewhat higher for the decedents, though not significantly (71.4% vs. 54.8%, p = 0.146, 37.1% vs. 26%, p = 0.236 and 68.6% vs. 52.1%, p = 0.104 respectively).

### Biomarkers

The majority of patients (90, 83.3%) had CFD levels above the cutoff level of 850 ng/ml. We found a significantly higher level of CFD taken upon admission in patients who died to those who survived: median 3469.0 (IQR 1984.0–8833.0) vs. 1659 (IQR 855.5–2613.5), p<0.001. PCT was measured in 70 (64.8%) subjects and again, higher levels of PCT were found non-survivor patients compared to those who survived: median 6.49 (IQR 0.91–13.87) vs. 1.05 (IQR 0.37–7.69), p = 0.06.

Lactate at admission was obtained for 97 (89.8%) patients and higher levels of lactate were found in non-survivor patients compared to those who survived: median 2.1 (IQR 1.25–4.3) vs. 1.2 (IQR 0.90-1.87), p = 0.007. There was a correlation between APACHE II score and CFD level (rho = 0.2, p = 0.041) and between lactate and CFD levels (rho = 0.28, p = 0.005). However, correlations between CFD and PCT, PCT and APACHE II score, lactate and APACHE II score, PCT and lactate, were not significant (rho = 0.14, p = 0.24; rho = 0.04, p = 0.77; rho = 0.16, p = 0.22; rho = 0.17, p = 0.10 respectively). Bacteremia was not associated with the higher levels of CFD: median 2469 (IQR 1220–4154) vs. 1947 (IQR 983.5–3186), p = 0.211.

### In-hospital or 28-day mortality

**Figure 1:**
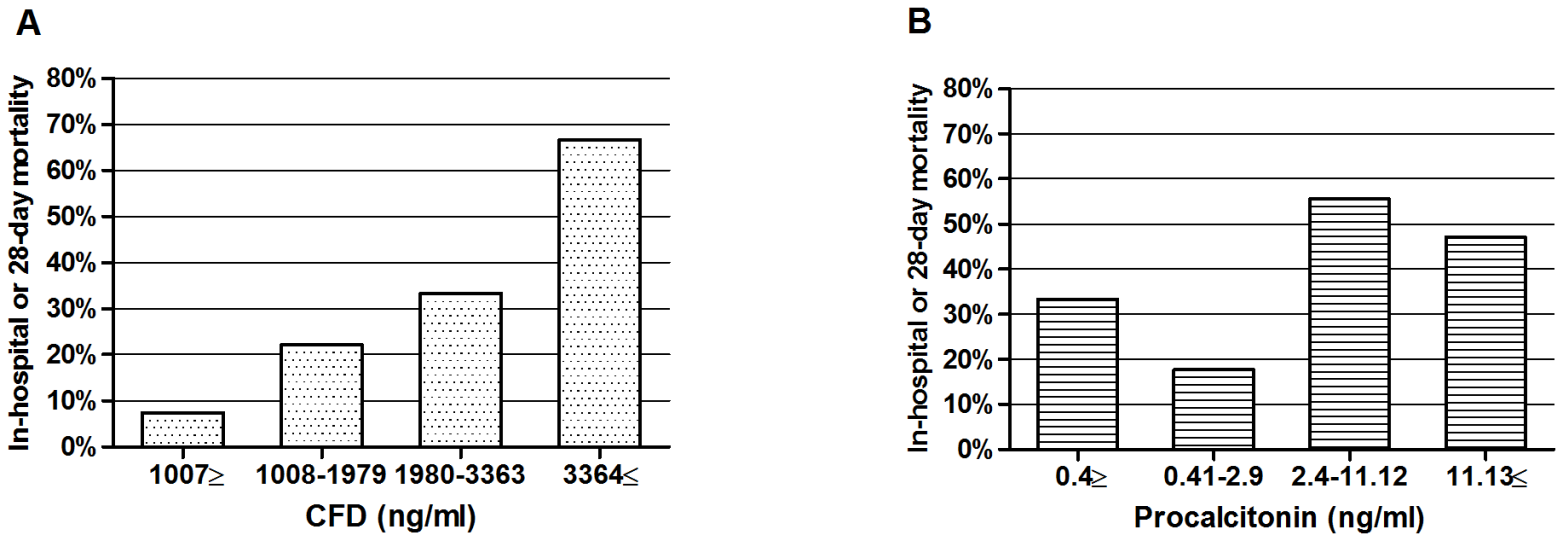
In-hospital or 28-day mortality percentage by CFD quartiles (A) and procalcitonin (PCT) quartiles (B) on hospital admission. CFD P-value for trend: p<0.001, PCT P-value for trend: p = 0.109.

The study group was divided into quartiles by CFD and PCT levels (Figure 1). The in-hospital or 28-day mortality rate was 7.4% in the lowest quartile and 66.7% in the highest quartile for CFD (p for trend <0.001), 33.3% in the lowest quartile and 47.1% in the highest quartile for PCT (p for trend  = 0.109). The study group was also divided into quartiles by lactate levels (not shown) with mortality rate of 21.4% in the lowest quartile and 54.2% in the highest quartile for lactate (p for trend = 0.015). The quartiles of CFD showed homogeneous ascent in mortality rates while the quartiles of PCT and lactate did not (figure 1).

**Figure 2:**
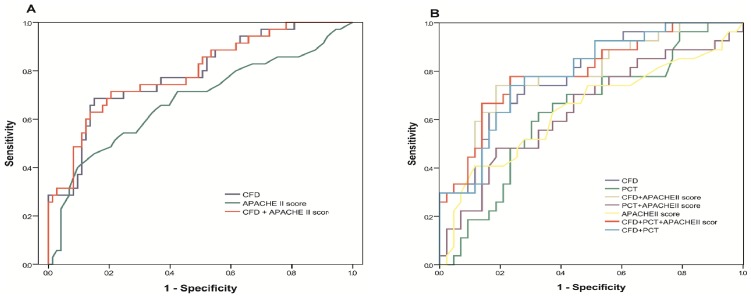
ROC curve of CFD levels and APACHE II score as predictors of in-hospital and 28-day mortality (A). **CFD** P-value for trend: p<0.001, **PCT** P-value for trend: p = 0.109. **Score: CFD**; AUC = 0.79 (0.69–0.68), P<0.05 compared to APACHE II, IDI = 0.14 (0.03–0.25). **APACHE-II+CFD**; AUC = 0.79 (0.70–0.89), P<0.05, IDI = 0.18 (0.09-0.28). **APACHE-II**; AUC = 0.68 (0.56–0.79), Reference. **ROC curve of CFD levels, PCT levels and APACHE II scores as predictors of in-hospital and 28-day mortality (B). Score: CFD**; AUC = 0.79 (0.68–0.90), P<0.05 compared to APACHE II, IDI = 0.16 (0.04–0.29). **PCT**; AUC = 0.63(0.50–0.77), P>0.05, IDI = −0.04 (0.01–0.10). **APACHE-II+CFD**; AUC = 0.80(0.68–0.90), P<0.05, IDI = 0.20(0.09–0.31). **APACHE-II+PCT**; AUC = 0.65(0.51–0.78), P>0.05, IDI = 0.01(0.01–0.02). **APACHE-II+CFD+PCT**; AUC = 0.80(0.69–0.91), P<0.05, IDI = 0.16(0.04–0.27). **CFD+PCT**; AUC = 0.79(0.68–0.90), P<0.05, IDI = 0.13(−0.01–0.26). **APACHE-II**; AUC = 0.64(0.50–0.78), Reference.

Table 2 presents logistic regression models adjusted for APACHE II score, CFD levels divided into quartiles, PCT levels divided into quartiles and lactate levels divided into quartiles. APACHE II score and CFD were significantly associated with mortality, the later having a higher OR per quartile increase than the former (OR = 1.05, 95% CI 1–1.11, p = 0.049 vs. OR = 2.57, 95% CI 1.61–4.08, p<0.001). C-statistics for 28-day or in-hospital mortality models was 0.79 (95% CI 0.69–0.88) for CFD and 0.68 (95% CI 0.56–0.79) for APACHE II on admission (Figure 2a, p<0.001). IDI analyses shows that CFD and CFD+APACHE II score models had better discriminatory ability for the mortality prediction than APACHE II score alone.

**Table 2:** Logistic regression model of in-hospital and 28-day mortality in patients with severe sepsis.

	Odds ratio	95% CI	p-value
**N = 108**			
**APACHE II score**	1.05	1–1.11	0.049
**CFD (quartiles)**	2.57	1.61–4.08	<0.001
**N = 70**			
**APACHE II score**	1.04	0.98–1.11	0.226
**CFD (quartiles)**	2.49	1.48–4.19	0.001
**Procalcitonin (PCT, quartiles)**	1.39	0.84–2.31	0.2
**N = 97**			
**APACHE II score**	1.05	0.99–1.11	0.085
**CFD (quartiles)**	2.39	1.42–4.04	0.001
**Lactate (quartiles)**	1.41	0.90–2.22	0.136

In subgroup of 70 patients with both CFD and PCT biomarkers available (Table 2) CFD remained significant while PCT was not (OR = 2.49, 95% CI 1.48–4.19, p = 0.001 vs. OR = 1.39, 95% CI 0.84–2.31, p = 0.2). C-statistics for mortality models was 0.79 (95% CI 0.68–0.90) for CFD, 0.63 (95% CI 0.50–0.80) for PCT and 0.64 (95% CI 0.50–0.78) for APACHE II score (Figure 2b, p<0.001). IDI analyses shows that in comparison to APACHE II score, CFD remained a strong discriminator while PCT did not differ from APACHE II score. Model with APACHE II and CFD levels had the highest discriminatory ability for mortality prediction.

In subgroup of 97 patients with CFD and lactate biomarkers available (Table 2) CFD quartiles remained significant predictor of mortality (OR = 2.39, 95% CI 1.42–4.04, p = 0.001), while lactate was not (OR = 1.41, 95% CI 0.90–2.22, p = 0.136). IDI analyses shows that in comparison to APACHE II score, lactate had a similar discriminatory ability: IDI = 0.03 (95% CI −0.06–0.12, p = 0.45).

**Figure 3:**
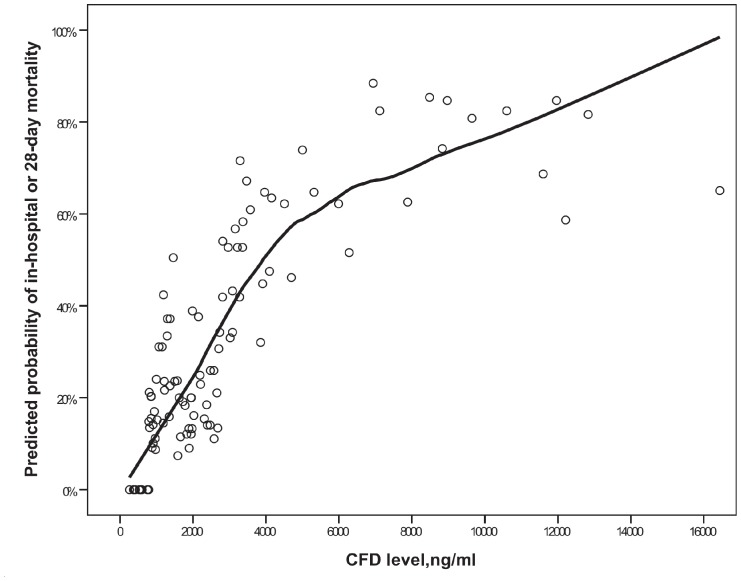
Locally weighted polynomial regression (LOESS) analysis for CFD based probability of in-hospital or 28-day mortality adjusted for APACHE II score on hospital admission.

Figure 3 depicts LOESS analysis. The curve depicts predicted probability for death depending on the CFD level adjusted for APACHE II score. It reveals a steady monotonic increase in mortality risk with increasing CFD levels.

## Discussion

In this study we have shown that CFD admission levels have good accuracy and precision in predicting short term mortality in patients with severe sepsis admitted to the intensive care. CFD surpassed both the clinical score (APACHE –II) and PCT in its ability to discriminate between 28 days survivors and decedents.

Despite the progress in advanced treatment options, severe sepsis continues to be a disease with a high mortality rate [20], [21], [22], [23]. With the aging of Western populations, the success in treatment and prevention of previously lethal cardiovascular and infectious diseases, and improvements in preventive medicine, the incidence of sepsis and severe sepsis, as a major common pathway for end-of-life events, has increased and is expected to increase further [24].

In era of searching for new biomarkers which can provide early diagnosis and management of sepsis patients, CFD assay becomes focus of interest. A recent editorial related to use of CFD assays pointed out that CFD seemed to be among the most promising prognostic markers that could be used in severe sepsis [25]. However, quantitative PCR, the most widely used technique for assessing CFD levels is costly and labor- intensive. PCR requires fast separation of blood and storage of plasma at -80°C to avoid DNA fragmentation. In addition, this assay measures copy number of the gene under investigation and not DNA concentration, and it is affected by loss from extraction, by DNA fragmentation and PCR efficiency. In contrast, measurement of DNA concentration by our assay requires only the mixture of a small serum or plasma sample with a fluorescent dye; collected blood can be kept at room temperature for few hours, no extraction or incubation are needed and results are promptly available [15]. Our assay was used in previous clinical studies by us [5], [8], [15], [18], [26] and others [27], [28].

Using our new simple method we have shown that patients who died during hospitalization or in 28 days had elevated admission CFD levels compared to survivors, while PCT levels were not significantly different between the two groups. In addition, CFD divided into quartiles was associated with gradual increase in mortality changing from 7.4% in the low to 66.7% in the upper quartiles. Furthermore, LOESS analysis evaluating the relationship between admission CFD level and the predicted APACHE adjusted death probability showed almost a linear association.

CFD was previously measured by various techniques to assess its prognostic value for patients admitted to intensive care units [6], [9], [10], [11], [12], [13], [14], [29]. Lo et al. found that CFD was an effective marker for complications and mortality prediction [6]. Wijeratne et al. found that CFD was better than CRP for prediction of death in intensive care unit [9]. Rhodes et al. on 52 patients, Saukkonen et al. on a large cohort of 255 patients and Huttunen on 132 patients found that for septic patients CFD is an independent predictor of death [10], [11], [13]. Similar to our study Moreira et al. found elevated CFD in non-survivors septic patients comparing to survivors, while PCT and CRP were not significantly different between these two groups [12]. In recent study Dwivedi et al. showed that CFD had a better prognostic utility than APACHE II score [14].

We have shown that CFD betters PCT as a mortality predictor. We believe that the advantageous prognostic ability of CFD comparing to other serological marker, including PCT, derives from the integration in its value of the cumulative organ damage and systemic inflammation. The limitation of PCT and similar other inflammatory markers such as CRP and IL-6 is that they don't reveal ongoing necrosis and apoptosis and mainly reflect the levels of microbial products and proinflammatory cytokines [30]. Most cells contain the same amount of DNA, consequently the released of DNA from injured tissue is in linear association to the damage. An additional source of DNA under septic condition are activated neutrophils which release in response to the inflammatory storm a mixtures of DNA and nuclear proteins, named neutrophil extracellular traps (NETs), that form extracellular fibers that bind Gram-positive and -negative bacteria [31], [32]. Thus, the ability of CFD to accurately predict 28 days mortality probably derives from reflecting both ongoing cell death and inflammation that will cause future tissue damage.

The model based on combination of CFD with APACHE II had a better discriminatory ability than both scores alone as indicated by the improvement in IDI value. The APACHE II score combines inflammation markers such as fever and leukocytes count and markers of physiological impairment such as blood pressure and pH which are affected by organ dysfunction. The marked added value of CFD comes probably form the direct assessment of damage while PCT, an inflammatory marker, might have no added value beyond the APACHE II score.

Our study has several important limitations. This is a single center study with a relatively small sample size. PCT levels were measured only in 70 patients, further decreasing the analysis power. SOFA data are not provided: CFD was only measured only upon admission without follow-up. A large, multicenter, observational study is necessary to further validate our findings.

## References

[1] Knaus WA, Draper EA, Wagner DP, Zimmerman JE (1985) APACHE II: a severity of disease classification system. Crit Care Med 13: 818–829. 3928249

[2] Vincent JL, de Mendonca A, Cantraine F, Moreno R, Takala J, et al (1998) Use of the SOFA score to assess the incidence of organ dysfunction/failure in intensive care units: results of a multicenter, prospective study. Working group on “sepsis-related problems” of the European Society of Intensive Care Medicine. Crit Care Med 26: 1793–1800. 9824069 10.1097/00003246-199811000-00016

[3] Calfee CS, Pugin J (2012) The search for diagnostic markers in sepsis: many miles yet to go. Am J Respir Crit Care Med 186: 2–4. 22753680 10.1164/rccm.201205-0854ED PMC3400993

[4] Lichtenstern C, Brenner T, Bardenheuer HJ, Weigand MA (2012) Predictors of survival in sepsis: what is the best inflammatory marker to measure? Curr Opin Infect Dis 25: 328–336. 22421751 10.1097/QCO.0b013e3283522038

[5] Czeiger D, Shaked G, Eini H, Vered I, Belochitski O, et al (2011) Measurement of circulating cell-free DNA levels by a new simple fluorescent test in patients with primary colorectal cancer. Am J Clin Pathol 135: 264–270. 21228367 10.1309/AJCP4RK2IHVKTTZV

[6] Lo YM, Rainer TH, Chan LY, Hjelm NM, Cocks RA (2000) Plasma DNA as a prognostic marker in trauma patients. Clin Chem 46: 319–323. 10702517

[7] Chang CP, Chia RH, Wu TL, Tsao KC, Sun CF, et al (2003) Elevated cell-free serum DNA detected in patients with myocardial infarction. Clin Chim Acta 327: 95–101. 12482623 10.1016/s0009-8981(02)00337-6

[8] Tovbin D, Novack V, Wiessman MP, Elkadir AA, Zlotnik M, et al (2012) Circulating cell-free DNA in hemodialysis patients predicts mortality. Nephrol Dial Transplant 27: 3929–3935. 22833622 10.1093/ndt/gfs255

[9] Wijeratne S, Butt A, Burns S, Sherwood K, Boyd O, et al (2004) Cell-free plasma DNA as a prognostic marker in intensive treatment unit patients. Ann N Y Acad Sci 1022: 232–238. 15251966 10.1196/annals.1318.036

[10] Rhodes A, Wort SJ, Thomas H, Collinson P, Bennett ED (2006) Plasma DNA concentration as a predictor of mortality and sepsis in critically ill patients. Crit Care 10: R60. 16613611 10.1186/cc4894 PMC1550922

[11] Saukkonen K, Lakkisto P, Pettila V, Varpula M, Karlsson S, et al (2008) Cell-free plasma DNA as a predictor of outcome in severe sepsis and septic shock. Clin Chem 54: 1000–1007. 18420731 10.1373/clinchem.2007.101030

[12] Moreira VG, Prieto B, Rodriguez JS, Alvarez FV (2010) Usefulness of cell-free plasma DNA, procalcitonin and C-reactive protein as markers of infection in febrile patients. Ann Clin Biochem 47: 253–258. 20421309 10.1258/acb.2010.009173

[13] Huttunen R, Kuparinen T, Jylhava J, Aittoniemi J, Vuento R, et al (2011) Fatal outcome in bacteremia is characterized by high plasma cell free DNA concentration and apoptotic DNA fragmentation: a prospective cohort study. PLoS One 6: e21700. 21747948 10.1371/journal.pone.0021700 PMC3128600

[14] Dwivedi DJ, Toltl LJ, Swystun LL, Pogue J, Liaw KL, et al (2012) Prognostic utility and characterization of cell-free DNA in patients with severe sepsis. Crit Care 16: R151. 22889177 10.1186/cc11466 PMC3580740

[15] Goldshtein H, Hausmann MJ, Douvdevani A (2009) A rapid direct fluorescent assay for cell-free DNA quantification in biological fluids. Ann Clin Biochem 46: 488–494. 19729503 10.1258/acb.2009.009002

[16] Tschaikowsky K, Hedwig-Geissing M, Braun GG, Radespiel-Troeger M (2011) Predictive value of procalcitonin, interleukin-6, and C-reactive protein for survival in postoperative patients with severe sepsis. J Crit Care 26: 54–64. 20646905 10.1016/j.jcrc.2010.04.011

[17] Levy MM, Fink MP, Marshall JC, Abraham E, Angus D, et al (2003) 2001 SCCM/ESICM/ACCP/ATS/SIS International Sepsis Definitions Conference. Crit Care Med 31: 1250–1256. 12682500 10.1097/01.CCM.0000050454.01978.3B

[18] Shimony A, Zahger D, Gilutz H, Goldstein H, Orlov G, et al (2010) Cell free DNA detected by a novel method in acute ST-elevation myocardial infarction patients. Acute Card Care 12: 109–111. 20712451 10.3109/17482941.2010.513732

[19] Pencina MJ, D'Agostino RB Sr, D'Agostino RB Jr, Vasan RS (2008) Evaluating the added predictive ability of a new marker: from area under the ROC curve to reclassification and beyond. Stat Med 27: 157–172 discussion 207–112. 17569110 10.1002/sim.2929

[20] Angus DC, Linde-Zwirble WT, Lidicker J, Clermont G, Carcillo J, et al (2001) Epidemiology of severe sepsis in the United States: analysis of incidence, outcome, and associated costs of care. Crit Care Med 29: 1303–1310. 11445675 10.1097/00003246-200107000-00002

[21] Bernard GR, Vincent JL, Laterre PF, LaRosa SP, Dhainaut JF, et al (2001) Efficacy and safety of recombinant human activated protein C for severe sepsis. N Engl J Med 344: 699–709. 11236773 10.1056/NEJM200103083441001

[22] Rivers E, Nguyen B, Havstad S, Ressler J, Muzzin A, et al (2001) Early goal-directed therapy in the treatment of severe sepsis and septic shock. N Engl J Med 345: 1368–1377. 11794169 10.1056/NEJMoa010307

[23] van den Berghe G, Wouters P, Weekers F, Verwaest C, Bruyninckx F, et al (2001) Intensive insulin therapy in critically ill patients. N Engl J Med 345: 1359–1367. 11794168 10.1056/NEJMoa011300

[24] Kumar G, Kumar N, Taneja A, Kaleekal T, Tarima S, et al (2011) Nationwide trends of severe sepsis in the 21st century (2000–2007). Chest 140: 1223–1231. 21852297 10.1378/chest.11-0352

[25] Rhodes A, Cecconi M (2012) Cell-free DNA and outcome in sepsis. Crit Care 16: 170. 23140420 10.1186/cc11508 PMC3672553

[26] Ohayon S, Boyko M, Saad A, Douvdevani A, Gruenbaum BF, et al (2012) Cell-free DNA as a marker for prediction of brain damage in traumatic brain injury in rats. J Neurotrauma 29: 261–267. 22149927 10.1089/neu.2011.1938

[27] Kohlova M, Ribeiro S, do Sameiro-Faria M, Rocha-Pereira P, Fernandes J, et al (2013) Circulating cell-free DNA levels in hemodialysis patients and its association with inflammation, iron metabolism, and rhEPO doses. Hemodial Int 17: 664–667. 23679088 10.1111/hdi.12055

[28] Coimbra S, Catarino C, Costa E, Oliveira H, Figueiredo A, et al (2014) Circulating cell-free DNA levels in Portuguese patients with psoriasis vulgaris according to severity and therapy. Br J Dermatol 170: 939–942. 24245854 10.1111/bjd.12738

[29] Okkonen M, Lakkisto P, Korhonen AM, Parviai-nen I, Reinikainen M, et al (2011) Plasma cell-free DNA in patients needing mechanical ventilation. Crit Care 15: R196. 21838858 10.1186/cc10357 PMC3387638

[30] Riedel S (2012) Procalcitonin and the role of biomarkers in the diagnosis and management of sepsis. Diagn Microbiol Infect Dis 73: 221–227. 22704255 10.1016/j.diagmicrobio.2012.05.002

[31] Brinkmann V, Reichard U, Goosmann C, Fauler B, Uhlemann Y, et al (2004) Neutrophil extracellular traps kill bacteria. Science 303: 1532–1535. 15001782 10.1126/science.1092385

[32] McDonald B, Urrutia R, Yipp BG, Jenne CN, Kubes P (2012) Intravascular neutrophil extracellular traps capture bacteria from the bloodstream during sepsis. Cell Host Microbe 12: 324–333. 22980329 10.1016/j.chom.2012.06.011

